# Stability of the emulsion during the injection of anionic and cationic surfactants in the presence of various salts

**DOI:** 10.1038/s41598-023-38428-8

**Published:** 2023-07-13

**Authors:** Hamid Garmsiri, Shahriar Jahani, Yousef Kazemzadeh, Mohammad Sharifi, Masoud Riazi, Reza Azin

**Affiliations:** 1 Department of Petroleum Engineering, School of Chemical and Petroleum Engineering, Shiraz University, Shiraz, Iran; 2 Enhanced Oil Recovery (EOR) Research Centre, IOR EOR Research Institute, Shiraz University, Shiraz, Iran; 3 Department of Petroleum Engineering, Faculty of Petroleum, Gas, and Petrochemical Engineering, Persian Gulf University, Bushehr, Iran; 4 Persian Gulf University-Northeast Petroleum University of China Joint Research Laboratory, Oil and Gas Research Center, Persian Gulf University, Bushehr, Iran; 5 Department of Petroleum Engineering, Amirkabir University of Technology, Tehran Polytechnic, Tehran, Iran

**Keywords:** Engineering, Chemical engineering

## Abstract

Smart water injection is one of the engineering techniques to enhance oil recovery (EOR) from carbonate and sandstone reservoirs that have been widely used in recent decades. Wettability alteration and IFT are among the essential and influential mechanisms that can be mentioned to achieve EOR. One of the critical issues in the field of EOR is the effect of reservoir ions on the formation and stability of the emulsion. Investigating the role and performance of these ions during EOR processes is of significant importance. These processes are based on smart water injection and natural production. In this research, stability was investigated and formed during the injection of different concentrations of anionic and cationic surfactants, respectively alpha olefin sulfonate (AOS) and cetrimonium bromide (CTAB), into a water–oil emulsion with a volume ratio of 30–70. Considering the droplet diameter distribution and the flow speed of separation by centrifugation, the optimal concentration level has been investigated in both surfactants. Based on the results, the highest stability and emulsion formation occurred in the presence of AOS surfactant. Then different concentrations of CaCl_2_, MgCl_2_, and NaCl salts were added in optimal concentrations of both surfactants. The formation and stability of the emulsion was checked by examining the distribution of the droplet diameter and the separation flow rate. AOS anionic surfactant had the most stability in the presence of MgCl_2_ salt, and better performance in stability of the emulsion was obtained. The maximum number of droplet diameters in the optimal concentration for AOS and CTAB surfactant systems is 1010 and 880, respectively, and for binary systems of AOS surfactant and MgCl_2_, CaCl_2_ and NaCl salts, it is 2200, 1120 and 1110, respectively. Furthermore, for the CTAB binary system in the presence of MgCl_2_, CaCl_2_, and NaCl salts, it is 1200, 1110, and 1100, respectively. The stability of the emulsion of salts in the presence of both AOS and CTAB surfactants was MgCl2 > CaCl_2_ > NaCl.

## Introduction

Production from hydrocarbon reservoirs is generally divided into three stages, which include primary, secondary, and tertiary production. Primary production includes the production of oil by the natural forces of the reservoir. After the primary production, the reservoir pressure drops, and methods such as water or gas injection must be used as secondary recovery to raise or maintain the reservoir pressure^1–3^. It has been found that despite the injection of water and the recovery of the initial lost pressure of the reservoir, there has not been much change in the amount of oil production in the reservoir, so we need to use the methods of increasing or tertiary production^4–6^. The definition of enhanced oil recovery (EOR) is the use of various techniques and methods to increase oil production by applying energy from outside or injecting materials into reservoirs that are not capable of economic production under their natural conditions. During water injection, the chemical and physical characteristics of the injected water are of particular importance, which has a significant effect on the wettability of the reservoir rock, changes in the surface tension between the injected water and oil, and finally, the production of oil from the reservoir^7,8^. Investigating the rate of formation and stability of emulsions is one of the essential things studied and investigated in the last few decades. The formation of emulsion in reservoir conditions can be considered one of the new methods of EOR. During the processes related to the EOR through the injection of smart water into hydrocarbon reservoirs, the emulsion formation is one of the common phenomena that we will face. In the topic of emulsion formation and stability, there are different and many factors that influence the degree of stability and its formation, which, based on the amount and type of substance in the system, can help to form and improve its stability or prevent it from forming. The concentration of emulsifiers is one of the decisive and vital factors in the formation and stability of emulsions^9^. In this case, water injection is considered one of the tertiary processes for oil production. The resulting investigations also indicate that ions such as Ca^2+^, Mg^2+^, Na^2+^, and ${\text{So}}_{4}^{2 - }$, which are known as “potential determinants ions”, have a positive effect on oil recovery^10,11^. These ions can cause changes in the oil and water system due to their charge and ionic radius, or in other words, their proper charge density. Different ions will have different IFT when they are present in water, and these ions will also have changes in IFT alterations. The specified concentrations of these active ions must be at a suitable level so that they can attract the polar parts of the oil towards the interface between the two fluids and also change the arrangement in such a way that the IFT tends to decrease, and also reduce the available capillary force and finally increase the desired oil recovery^12,13^. The presence of active ions can make it possible to form all kinds of emulsions, which in some cases improve production and, in others, reduces it. Based on this, the investigation of ions and surfactants in forming emulsions resulting from flooding is also of particular importance^14,15^.

The extraordinary properties of aqueous surfactant solutions can be attributed to a hydrophilic radical head and a hydrophobic (lipophilic) chain in the molecule. The polar or ionic head of the radical is attracted to the aqueous environment. These dissolutions take place by ion–dipole or dipole–dipole adsorptions. Naturally, the polar head used to distributes the surfactants in different categories^16,17^. In general, surfactants are divided into four types; Anionic is used in sandstones due to the same polar charge of surfactant and rock surface, cationic is used in carbonate reservoirs, non-ionic is used in both types of carbonate rock and sandstone, but depends on the effect of oil recovery to the trap, it can be used after flooding with secondary water, and finally, the amphoteric type, which is both expensive and rare^18,19^. The statistical analysis of the dimensions of the emulsion droplets can give us a good view of the efficiency of the emulsification process. Observing changes in particle size distribution, such as the duration of emulsion formation, gives information about stability, which can be considered a unique characteristic effect of the same emulsion^20,21^. So, the most important indicator that can be considered for stability of the emulsion is the droplet size distribution because it controls the coagulation and aggregation of the droplets. For example, if two emulsions have the same average droplet size, they will behave differently due to the difference in droplet size distribution^22^. This problem is due to the control of stability of the emulsion through size distribution, which, if measured with time, can be an indicator to express stability, and emulsions with smaller droplets will have higher stability^23,24^. The possible mechanisms for the oil-in-water emulsion are:

1. Drops of oil through rock pores divert the flow towards the production well and improve oil recovery.
2. Oil dispersed in water is produced through the production of the water phase^25,26^.

On the other hand, for the emulsion of water in oil, the high viscosity also causes the production of oil, which is due to close the path and water channels. Also, based on the concept of Ganglion dynamics through the trapping of emulsion droplets in the pore throats, it requires a critical capillary force to move it. This is provided by increasing the viscous force by the continuous phase flow. Capillarity critical force refers to the force that causes the movement of the oil drop stuck in the pores. If the force is below the critical point, the trapped oil will increase the oil sweep by the aqueous continuous phase. Also, the injection of alkaline into the reservoir through emulsion formation causes an EOR^27,28^. The improved recovery when the water-in-oil emulsion is formed is due to increasing the mobility- ratio. By injecting alkaline materials into the reservoir, the production potential increases, especially heavy oil reservoirs, and by simultaneously reacting with the acidic components of heavy oil, through various mechanisms such as IFT alteration, emulsion formation, adhesion to the surface occurs, and the trapping of excess extraction takes place^29–31^. The predictive research that was carried out on water quality and the corrections needed to optimize the impact of water injected into the reservoirs is known as smart water^32^.

The necessary corrections that are usually made according to the type and requirement of the reservoir on the injected water include the following^33,34^:

1. Reducing the salinity of the water injected into the reservoir (such as seawater or water with low salinity)
2. Increasing the concentration of multivalent surface-active ions such as Ca^2+^ and ${\text{So}}_{4}^{2 - }$
3. Reducing the concentration of inactive surface ions such as Na^+^ and Cl^-^

Due to its abundance, low cost, and easy access, seawater is used as the primary source of water supply required for injection into reservoirs. In addition, due to the low salinity of seawater compared to formation water and the presence of active surface ions, it is a suitable option for obtaining modified water^35–37^.

The presence of different chemicals causes changes in the stability of the emulsion. The primary purpose of this study is to investigate the behaviour of different chemicals on the formation and stability of the emulsion. In the first part of the research, we will discuss the effect of surfactant concentration and salt concentration on the stability of the emulsion. Finally, in the next section, we will discuss the behaviour of the emulsion due to the presence of water percentage.

One of the research about surfactants and emulsions was done by Ghajavandi and Noor Mohammad in 2014. They used sodium dodecyl sulfate and hexadecyltrimethylammonium bromide during the EOR process of carbonated rock and concluded the surfactants increase the saline water injection rate and subsequently increase the production rate. They also observed that the addition of anionic surfactant does not have a significant effect on increasing the rate of absorbing hot water containing ions that affect wettability. They concluded that cationic surfactant has a higher potential to remove oil from oil-philic carbonate rock than anionic type^38^. Elyari et al., by investigating the movement behaviour of emulsions in pipelines, found that in high salinities, when water-in-oil emulsions are formed, the higher the salt concentration, the more stability is achieved^39^. In 2016, Abu Bakr et al., by researching the stability and behaviour of water-in-oil emulsions in different volume ratios, showed that in a high volume percentage of water (about 50%), the stability decreased, and also in low percentages of water, emulsion exhibits Newtonian behaviour^40^. In 2017, Maaref et al. used a micromodel to visually study the effect of different salts on the formation and stability of emulsions. The obtained results indicated that if it is possible to form an unstable emulsion, the measurable pressure difference at the two ends of the micromodel will face severe changes and fluctuations, which is due to the occurrence of the Snap-off mechanism^41^. Furthermore, in 2018, Maaref et al. the stability of water-in-oil emulsion were investigated by examining four different types of seawater, and the results showed that with the increase in sodium chloride concentration in seawater, the emulsion stability decreases due to the increase in droplet diameter. It was also stated that to form a stable emulsion, two changes in the direction of reducing the IFT and increasing the fluid's viscosity are among the requirements^42^. In 2018, Pal et al. The phase behaviour of micro-emulsion systems comprised of methyl ester sulfonate (MES) as a surfactant,1- propanol as a co-surfactant, and brine and alkane oils with varying chain lengths were studied for application in enhanced oil recovery. The prepared micro-emulsions were characterized by dynamic light scattering analysis, and the particle sizes have been obtained in the range of 5–80 nm. It was found that the injected microemulsion formulations can achieve about 30% oil recovery close to conventional secondary water-flooding^43^. In a 2018 study by Kumar et al., surfactants were screened based on their ability to reduce IFT values for proper formulation of stable nanoemulsions. Nanoemulsions prepared by high energy method using Tween 80 and n-heptane with droplet size in the 91.05–40.16 nm manifest kinetic stability due to continuous Brownian motion of droplets. The synergetic effect of silica nanoparticles (NPs) in the presence of Tween 80 shows enhancement in stability of nanoemulsion with the drop in droplets size from 36.15 to 21.37 nm and surface charge of − 59.05 mV. The formulated nanoemulsions show Pseudo-plastic behaviour with viscosity in the range of 9–12.5 mPa-s, which is further improved in the presence of nanoparticles^44^. In another study by Kumar et al. in 2019, microemulsion formulation was investigated using zwitterionic surfactant as amphiphile and mineral oil as the oleic phase. The optimized microemulsion obtained using RSM was characterized by particle size and zeta potential analysis. The stability and miscibility tests of microemulsion showed excellent results for applicability in chemical EOR. The core flooding experiment showed that the optimized microemulsion could recover 26.83% of additional oil as compared to 20.01% of oil recovery by surfactant flooding, over conventional water flooding^45^.

In 2019, Kazemzadeh et al. investigated the effect of asphaltene and different ions at different pressures and salinities on the stability of the emulsion, based on which the type and concentration of ions in smart water have a significant effect on the stability of the emulsion^9^.

Considering the importance of EOR in today’s world, this research aims is to determine and investigate the effect of smart water containing the simultaneous presence of salts and surfactants on the stability of the water-in-oil emulsion. One of the innovations that are the basis of this research and differentiate it from previous studies is using surfactant as an agent to form an emulsion. Also, in this research, for the first time, the optimal concentrations of two surfactants, AOS and CTAB and their effect on the stability and formation of water-in-oil emulsion in the presence and absence of monovalent and divalent salts such as NaCl, MgCl_2_ and CaCl_2_ and their comparison with Each other was paid.

AOS is an anionic surfactant, and CTAB is a cationic surfactant^46^. In this research, we will investigate the stability behaviour and formation of water-in-oil emulsions in the presence of anionic and cationic surfactants. It is possible that each of these surfactants may form a different oil-in-water emulsion than the water-in-oil emulsion. The purpose of this study is only to investigate water-in-oil emulsion.

In this research, for the first time, we compared two surfactants, while in other papers^47,48^, the influence of the behaviour of only one surfactant on the stability of the emulsion was discussed. Moreover, the innovation of this research was the synergistic effect of both surfactants and salts together. The measurement of their droplet diameter and its effect on the stability of the emulsion was investigated.

## Materials and Methods

The petroleum phase used in all experiments is normal industrial heptanes, and in the aqueous phase, distilled water made by Zolal Company was used. Also, to make the salts pure and reduce possible errors, salts from the German company Merck were used. To select the salts used in this research, the formation water analysis of two wells in Iran was used. In this analysis, the number of ions in the formation water was calculated in terms of parts per million and listed in Table 1.

**Table 1:** Analysis of ions present in the formation water of two wells in Iran.

Well	Na (ppm)	Ca (ppm)	Mg (ppm)	Fe (ppm)	Cl (ppm)	${\mathrm{SO}}_{4}$(ppm)	${\mathrm{HCO}}_{3}$(ppm)	TDS (ppm)	Salinity (ppm)
A-1	69,575	10,400	972	11	127,436	925	976	210,295	209,632
A-2	67,022	6000	729	67	115,299	986	342	190,445	189,667

As can be seen from the table above, the ions that include a higher percentage of formation water include the monovalent cation ion Na and two divalent cation ions Mg and Ca, and the monovalent anion ion Cl are available to a significant extent. By comparing the amounts of these ions, it can be seen that the Cl ion has relatively more amounts. The three selected salts will include a monovalent NaCl salt and two divalent salts, MgCl_2_ and CaCl_2_.

To determine the optimal concentration conditions for stable emulsion, the weight and volume of water and normal heptane should be measured first, which a digital scale with three decimal places is used, then surfactants and salt are added to the aqueous phase. After making the aqueous base solution with normal heptane, it was placed in a suitable container inside the shaker, which the maximum power of 500 rpm and the stirring time of the solution for 15 min were used, and the emulsion was formed.

**Figure 1:**
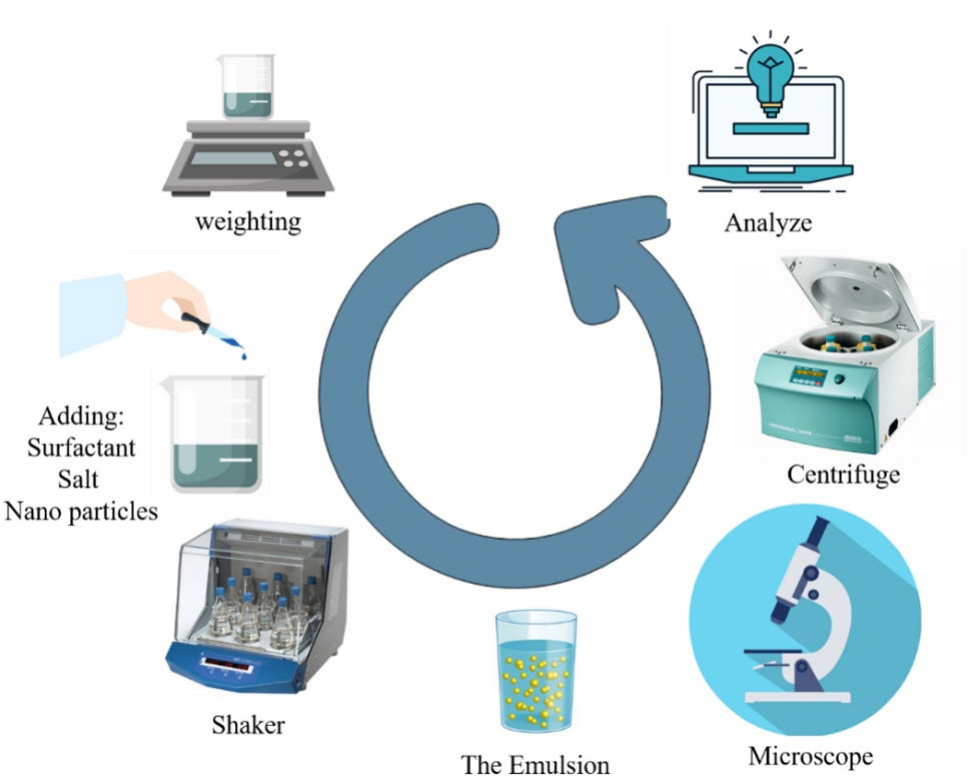
Schematic of the stages of the experiments.

Immediately after the end of the shaking device time, the sample will be taken under the microscope, and an image of the emulsion drop will be taken. The electronic microscope that will be used in this study is made by Nikon (model 200) and can magnify the image 100 times and save it through a memory card. Based on the need, the size and distribution of the formed emulsion droplets, required for photographing the formed sample, are selected. After imaging and determining the separation rate of the formed emulsion, the microscopic photo taken was transferred to the computer, and using the software for analyzing microscopic images (ImageJ), all the oil and water droplets visible in the image from several aspects, including the number and dimensions was studied and investigated. In the following, we calculate the separation rate of the emulsion using a centrifuge in terms of centrifuge revolutions per minute. The procedure of the tests is shown in Fig. 1.

### Microscopic photo analysis

Based on the microscopic photos prepared from the emulsion samples, it is possible to understand the droplet size distribution. Image-J software has been used to analyze these images and water dispersion in the continuous phase of normal heptane. To achieve correct and unequivocal results, microscopic photography of different places of the emulsion under the microscope has been prepared, and extraneous data are also removed to analyze the images accurately. If the emulsion does not have the desired stability and uniformity and significant differences are observed in different areas, photographs are taken from different points, and analysis is done by merging them.

**Figure 2:**
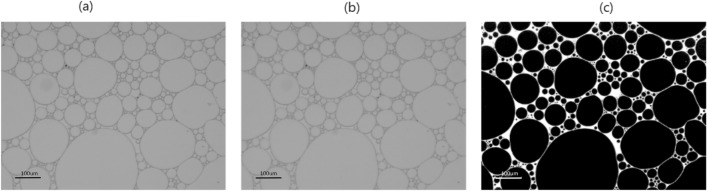
shows (**a**) the initial image of the emulsion, (**b**) the 8-bit image obtained from fluctuation correction, and (**c**) the number of emulsion droplets drawn by the software with 30% spherical accuracy.

Figure 2a shows a sample of a microscopic photo prepared from an emulsion. This image is given as an input to the ImageJ software, and converted into an 8-bit image like Fig. 2b, and the fluctuations are removed. To increase the accuracy of the software, the sphericity of the particles has been changed. It is considered to be 30%, and therefore the drops that do not meet this criterion are excluded from the analysis. Figure 2c shows the software's output as the number of emulsion droplets detected by the software. After statistical analysis, the mean droplet area indicates the stability of the emulsion. To calculate this parameter for the dispersed droplets of the aqueous phase inside the continuous oil phase, at least five photographs have been taken from each experiment. A number is reported under the title "mean droplet area." According to the results obtained, the maximum value of this parameter has been analyzed several times and has shown a difference of less than one square micrometre.

In this research, two types of surfactants, AOS and CTAB, and three salts, NaCl, MgCl_2_, and CaCl_2_, have been used in two experimental groups.

In the first experimental group, the aim is to investigate the effect of surfactant concentrations on the formation and stability of the emulsion, which was added to the aqueous phase at concentrations of 10,000, 5000, 1000, 500, and 100 (ppm), and then the emulsion (water-in-oil with the volume ratio of 30–70 emulsion have been studied) that was obtained in the shaker was put under the electron microscope. Also, the obtained emulsion is placed in the centrifuge to obtain the rotation rate in which the two phases are separated from each other.

In the experiments of the second group, after determining the optimal concentration for the two types of surfactants, for each of these surfactants, the values of the optimal concentrations of three salts NaCl, MgCl_2_, and CaCl_2_ are also calculated and determined. Therefore, at the optimal surfactant concentration determined in the previous step, each of the salts was added to the system in 7 concentrations of 100, 500, 1000, 5000, 10,000, 20,000, 60,000 (ppm), and again all the stages of the shaker device, microscopic imaging and centrifugation are also repeated for the prepared solutions. In this group, there are 3 types of salt, which include two divalent salts and one monovalent salt. In this experimental group, the aim is to compare monovalent and divalent cations, as well as the effect of different concentrations of salt on the stability of the emulsion was evaluated, and the dominant mechanisms are recognized.

The CMC measured with the oil phase sample for both AOS and CTAB surfactants was reported to be around 5000 ppm. Rheology increases up to 3 times the viscosity of the sample phase. The viscosity of water-in-oil emulsion increases up to 3 times compared to the viscosity of the oil phase sample. In the presence of different emulsions and stability, the viscosity may increase up to 3.5 times.

## Results and Discussion

In this research, as an innovation, the role of a natural emulsifier (i.e. asphaltene) has been removed and a synthetic emulsifier (i.e. surfactant) has been added to the system in question. For this purpose, unlike the previous studies where crude oil was used for the analysis of emulsion formation, in this study, normal heptane, which does not contain asphaltene, was used as the petroleum (non-polar) phase. Two types of surfactants, AOS and CTAB, have been used in this research. In the continuation of the first part, the results of the effect of the mentioned surfactants on the formation and stability of the emulsion are given.

In this study, the comparison of small droplets is being considered due to the significance of small emulsion droplets, which are fundamental to production processes from pores. Typically, the diameter of these pores is below twenty micrometers, thus necessitating an inquiry into emulsions with droplet diameters within this range.

### Examining the role of surfactant and its concentration in the formation and stability of the emulsion

To investigate the effect of surfactants in different concentrations, 100, 500, 1000, 5000, and 10,000 (ppm) of CTAB and AOS were added to distilled and normal heptane-water system and the results were compared. To check the formation or non-formation of the emulsion without the presence of surfactant, the two mentioned phases were placed next to each other and placed in a shaker for 15 min (500 rpm). The result obtained, as expected, indicated the absence of water-in-oil emulsion formation when the surfactant was not present in the aqueous phase.

#### The effect of AOS surfactant on the formation and stability of the emulsion

One of the goals determined in this research is to investigate and obtain the optimal concentration of surfactant. On the other hand, adding surfactant to the system, in addition to creating an emulsion, causes other changes such as a significant decrease in IFT, which is due to the high concentration of surfactant in the surface between the two phases.

**Figure 3:**
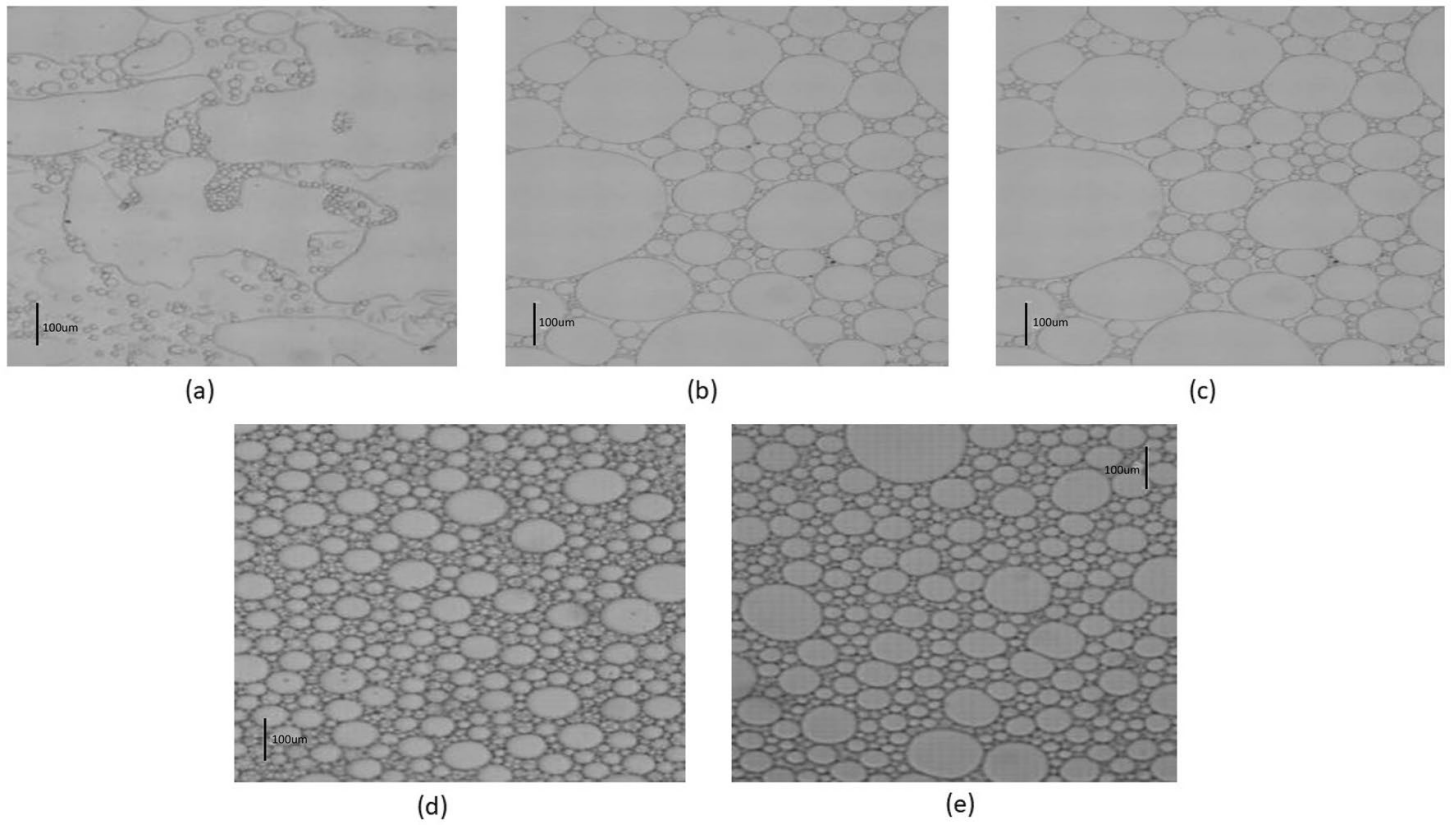
Microscopic images obtained from the emulsion formed in the system containing AOS surfactant.

When the water and oil phases are placed next to each other with specific volume percentages of 30 to 70% by weight respectively, if there is a sufficient amount of emulsifier agent between the two phases, by introducing mechanical force such as stirring or shaking, the aqueous (polar) phase will enter the continuous oil (non-polar) phase in the form of dispersed droplets and will become an emulsion. On the other hand, applying changes such as changes in the amount of surfactant also affected the quality of the formed emulsion. To investigate the effect of surfactant concentration on the formed emulsion, a solution is made in five separate containers at different concentrations mentioned and placed inside the shaking device. Figure 3 shows the microscopic images of emulsions obtained immediately after leaving the shaking device (and image concentration a: 100 ppm, b: 500 ppm, c: 1000 ppm, d: 5000 ppm, e: 10,000 ppm).

These images, which were prepared with a scale of 100 µm, show the effects of AOS surfactant concentration on emulsion formation. The visual results are in good agreement with the quantitative results and the quality of the formed emulsion has improved. The higher the number of dispersed phase droplets in the continuous phase and the smaller the droplet size, the better the stability of the formed emulsion. This stability means an increase in the resistance of the formed emulsion and the non-change of its properties against time.

**Figure 4:**
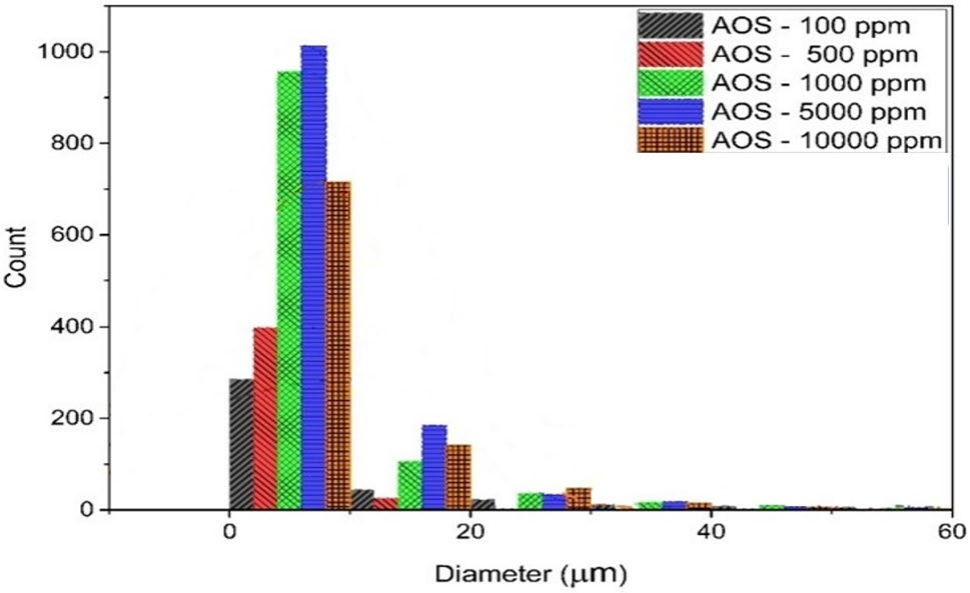
The droplet size distribution diagram of emulsion containing AOS surfactant.

Figure 4 is extracted by analyzing the microscopic photos obtained from the emulsion samples taken from the system containing AOS surfactant. In this diagram, to check the number and size of emulsion droplets, the particle size distribution diagram has been drawn for each of the samples.

According to Fig. 4, which shows the increase in the concentration of AOS surfactant from 100 to 5000 (ppm), the number of small droplets will increase greatly and the stability of the emulsion will increase. By passing the concentration of 5000 and increasing the concentration to 10,000 (ppm), the stability of the emulsions decreases, and the surfactants become colloidal and will not be present on the surface between the two phases. At the concentration of 5000 (ppm), the amount of stability and the number of small droplets are at their maximum compared to other concentrations, which makes it selected as the optimal concentration of surfactant.

**Figure 5:**
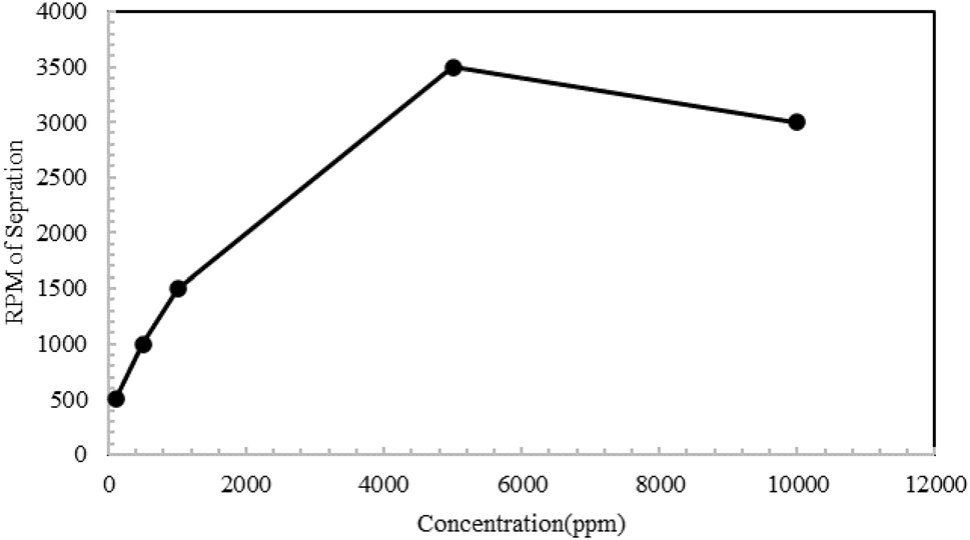
Required RPM from centrifuge to separate phases in normal heptane, water and AOS system.

To confirm and ensure this choice, another test was done using a centrifuge, which according to the diagram in Fig. 5, with the increase in the concentration of AOS surfactant, the smaller droplets of the emulsions increased and the stability increased. By increasing the concentration of surfactant up to 5000 (ppm), the separation rate of two phases increased up to 3500 rpm and after this concentration, if the amount of surfactant in the system increases, the stability level decreases and the separation rate decreases.

According to the results obtained from the analysis of microscopic images and the centrifuge device, it is concluded that the concentration of 5000 (ppm) is considered the optimal concentration of AOS surfactant, where the number of small droplets is maximum compared to other concentrations and it is separated at a higher cycle rate in the test of the centrifuge device.

#### Effect of CTAB surfactant on the formation and stability of the emulsion

Another surfactant used in the research is CTAB, which is a cationic surfactant. To check the ability of this surfactant in the formation and stability of the emulsion, like the AOS surfactant, this surfactant is added in concentrations of 5000, 1000, 500, 100, and 10,000 (ppm) to the deionized water and normal heptane system. Due to the lack of asphaltene in the system, which plays the role of a natural surfactant in the system, this synthetic surfactant acts as an emulsifier and causes the formation of an emulsion.

**Figure 6:**
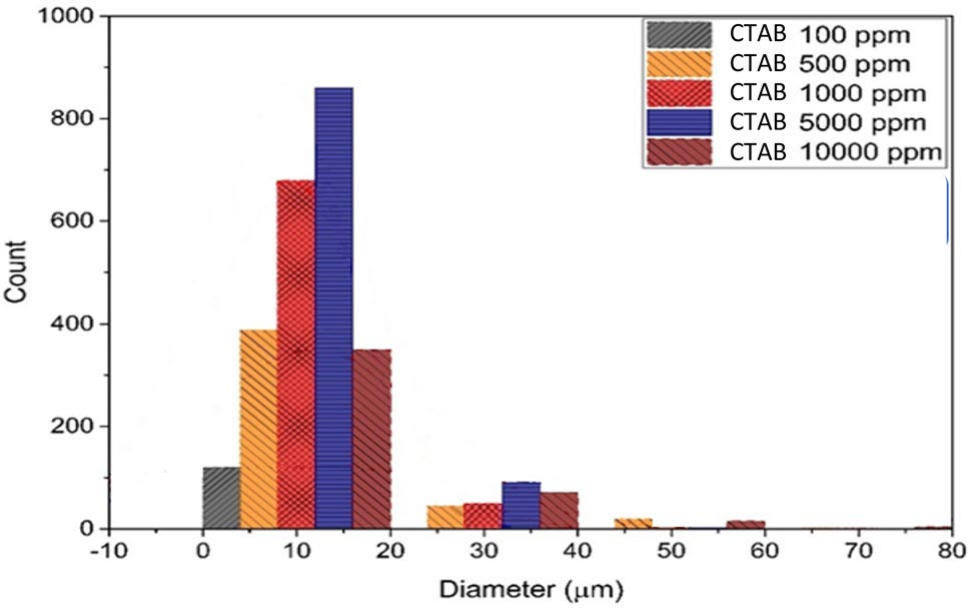
The droplet size distribution diagram of the emulsion containing CTAB surfactant.

Figure 6 shows the particle size distribution for CTAB surfactant. The way of emulsion formation for the system containing this surfactant is the same as that of AOS surfactant, and microscopic photography has been done after the shaking device. The resulting diagram of the distribution of emulsion droplets formed under the microscope will be as follows.

According to the distribution diagram of the frequency of emulsion droplets, as the amount of surfactant increases in the system, the number of small droplets has increased in a general trend. By increasing the surfactant concentration from 100 to 5000 (ppm), the stability of the emulsion has increased, which is the result of increasing the number of small droplets of the aqueous dispersed phase in the normal heptane continuous phase. In this diagram, with the increase in concentration from 5000 to 10,000 (ppm), the stability of the emulsion is greatly reduced and the emulsion particles are dispersed colloidally.

**Figure 7:**
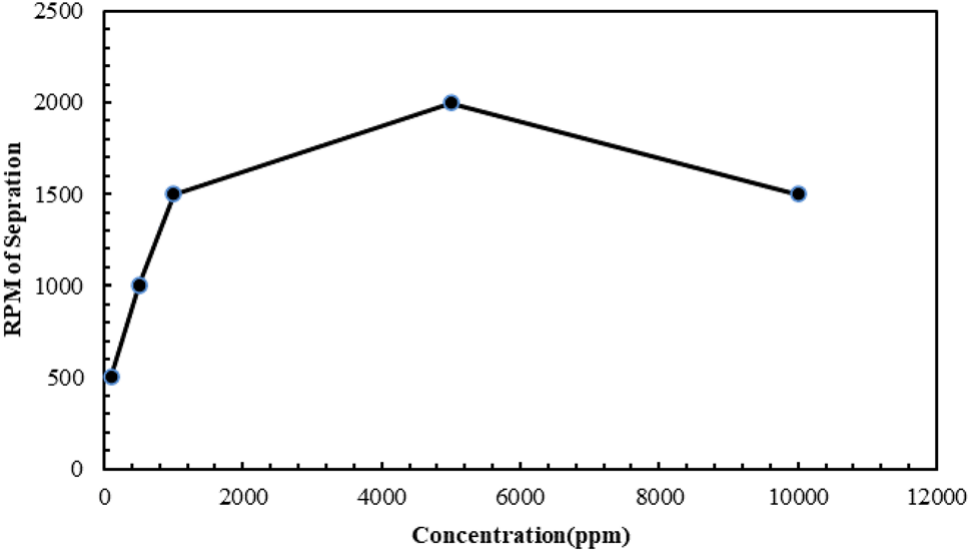
Centrifuge test results in the system containing normal heptane, water and CTAB surfactant.

Figure 7 shows the results obtained from the centrifuge test, which shows the rate of separation of the emulsion formed in the system containing CTAB.

Based on the drawn graph of the concentration of surfactant about the separation cycle rate of the formed emulsion, it is concluded that by increasing the amount of surfactant in the system up to a concentration of 5000 (ppm), the separation cycle rate (from at least 500 rpm) increased to 2000 rpm, which indicates an increase in the stability of the formed emulsion. After that, increasing the amount of surfactant in the system resulted in a decrease in the separation rate. Figures 5 and 7 show the maximum stability of the emulsion caused by the presence of surfactant at a concentration of 5000 (ppm). It should be mentioned that in the centrifuge test, the minimum rotation rate of the device is equal to 500 revolutions per minute, while it is not possible to set a lower rotation rate for the device, and it is possible that the separation rate of the emulsion with a concentration of 100 (ppm) was done in a value lower than 500 (rpm).

#### Comparison of AOS surfactant and CTAB surfactant in emulsion formation and stability

To check the stability and quality of two systems containing AOS and CTAB surfactants in the optimal concentration specified for each system, which was obtained by the droplet size distribution diagrams and the centrifuge, by comparing the number of small droplets and drawing the frequency distribution diagram, it is possible to check and select the steady state. By drawing the droplet frequency distribution diagram for the optimal state of two surfactants, it is concluded that the number of small emulsion droplets formed by AOS surfactant is more compared to CTAB surfactant, so the emulsion formed in the system containing AOS surfactant will be more stable.

**Figure 8:**
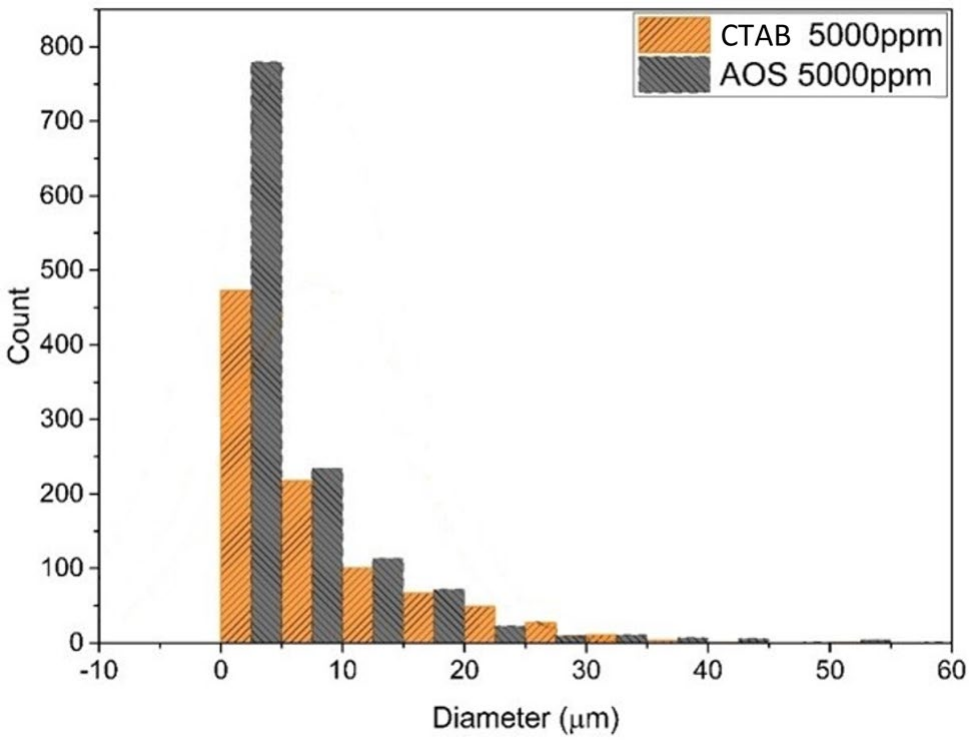
Comparison of droplet size distribution diagram of CTAB surfactant emulsion and AOS surfactant.

According to Fig. 8, the number of emulsion droplets with a radius smaller than 10 microns for the system containing AOS surfactant is about two times more than the results obtained with CTAB surfactant, which indicates the stability of the first system.

On the other hand, according to the rotation rates obtained from the experiments related to the centrifuge device to check the rotation rate of the separation of the two emulsions formed, for the CTAB surfactant at the optimal concentration of 5000 (ppm), 2000 rpm and for the AOS surfactant at the optimal concentration of 5000 (ppm), 3500 rpm was reported, which indicates the high rotation rate of the system containing AOS surfactant. Therefore, the emulsion formed in this system is more stable.

### Examining the role of salts and their concentration in the formation and stability of emulsion (in the presence of surfactant)

In this part of the research, the rate of formation and stability of emulsions in the conditions where the system contains salts of monovalent and divalent ions has been investigated. According to the previous part, the optimal conditions were determined in the presence of two types of anionic and cationic surfactants, and in the following, the effect of the presence of monovalent salt NaCl and bivalent MgCl_2_ and CaCl_2_ in the formation and stability of the formed emulsion was studied and investigated. According to the type of salts and the effect of loads and capacity of each, the optimal concentration of each may be different. The selection of salt has been done by examining the formation water of from two wells in Iran. These salts were added to the system in 7 different concentrations.

In this section, effect of different salts and surfactants in the stability of emulsion was studied. Table 2 Summarizes all experiments done at several salt concentrations for each salt in the presence of either CTAB or AOS surfactant. All experiments were conducted at the same temperature.

**Table 2:** Summary of experiments with the dual effect of surfactant and salt.

	Types systems	Surfactant concentration (ppm)	Salt concentrations (ppm)
1	NaCl–AOS	5000	100, 500, 1000, 5000, 10,000, 20,000
2	NaCl–CTAB	5000	100, 500, 1000, 5000, 10,000, 20,000, 60,000
3	CaCl_2_–AOS	5000	100, 500, 1000, 5000, 10,000, 20,000, 60,000
4	CaCl_2_–CTAB	5000	100, 500, 1000, 5000, 10,000, 20,000, 60,000
5	MgCl_2_–AOS	5000	100, 500, 1000, 5000, 10,000, 20,000, 60,000
6	MgCl_2_–CTAB	5000	100, 500, 1000, 5000, 10,000, 20,000, 60,000

#### NaCl–AOS system

**Figure 9:**
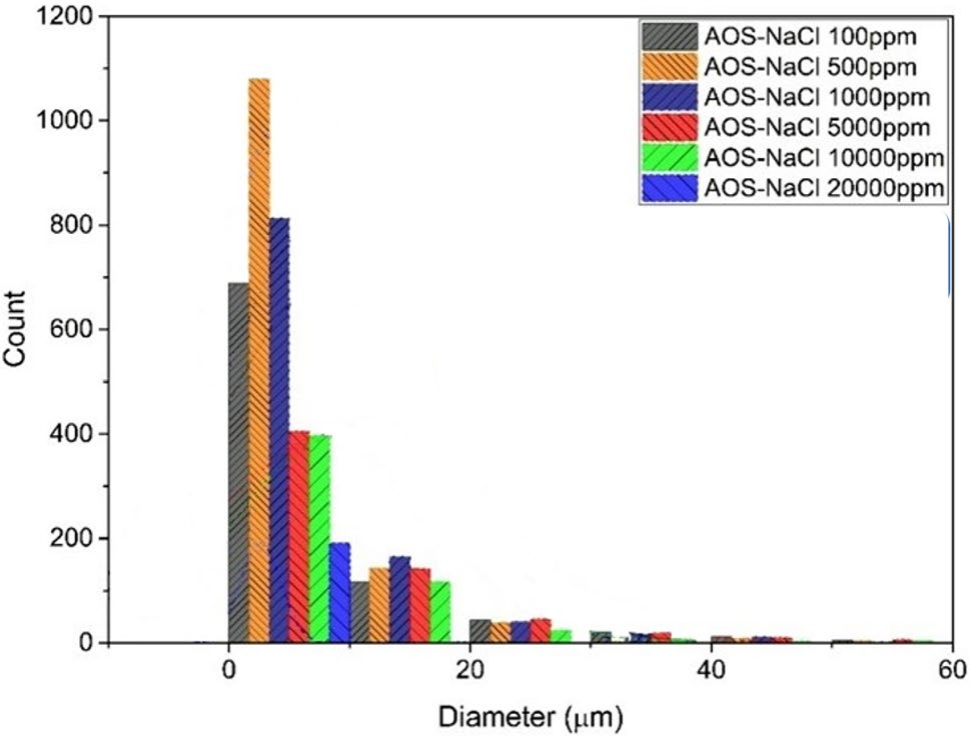
Droplet size distribution diagram of AOS surfactant emulsion and NaCl salt.

Figure 9 shows the stability of emulsion in the presence of NaCl salt and AOS surfactant. All experiments were conducted with the same surfactant concentration. According to this figure, maximum number of droplets were observed at the size of less than 10 µm which occurs at the 500 ppm concentration of the salt.

This salt has been added to the system containing a surfactant in seven concentrations of 100, 500, 1000, 5000, 10,000, 20,000, and 60,000 (ppm). Figure 9 shows the size distribution of emulsion droplets formed when NaCl salt is added to the surfactant-containing system. In general, if salt is added to the system, the number of small droplets is reduced, and by increasing the amount of salt present in the system up to a certain concentration, the stability of the formed emulsion is increased.

According to the number of changes in droplet size, with the increase of NaCl salt, the number of small drops increased with increasing concentration from 100 to 500 (ppm) and after this concentration, a decreasing trend was observed. In such a way that at the concentration of 60,000 (ppm) practically no emulsion was formed that could be photographed. When the salt concentration rises above a certain amount, it prevents the presence of surfactant in the contact surface of water and oil, therefore, preventing the presence of surfactant in the contact surface of water and oil reduces the stability of the emulsion.

Therefore, at the concentration of 500 (ppm), the number of microemulsion droplets formed is at its highest value, which somehow indicates the maximum level of stability in this particular concentration. To some extent, this phenomenon can be considered similar to Salt in effect and Salt out effect.

To obtain the optimal concentration separation rate for the system containing NaCl salt, the test of the results of the separation cycle rates has been designed. The stability of the emulsion is higher than this amount of salt concentration in the system, and the rate of separation rotation is reduced to 500 rpm, it should be noted that for the concentration of 60,000 (ppm) NaCl salt, no emulsion was formed in the laboratory vessel so that it is possible to check and measure the rate of rotation and separation. It is also possible that the separation cycle rate for other concentrations is less than 500 rpm, which is not possible to measure due to the limitation of the device.

#### NaCl–CTAB system

**Figure 10:**
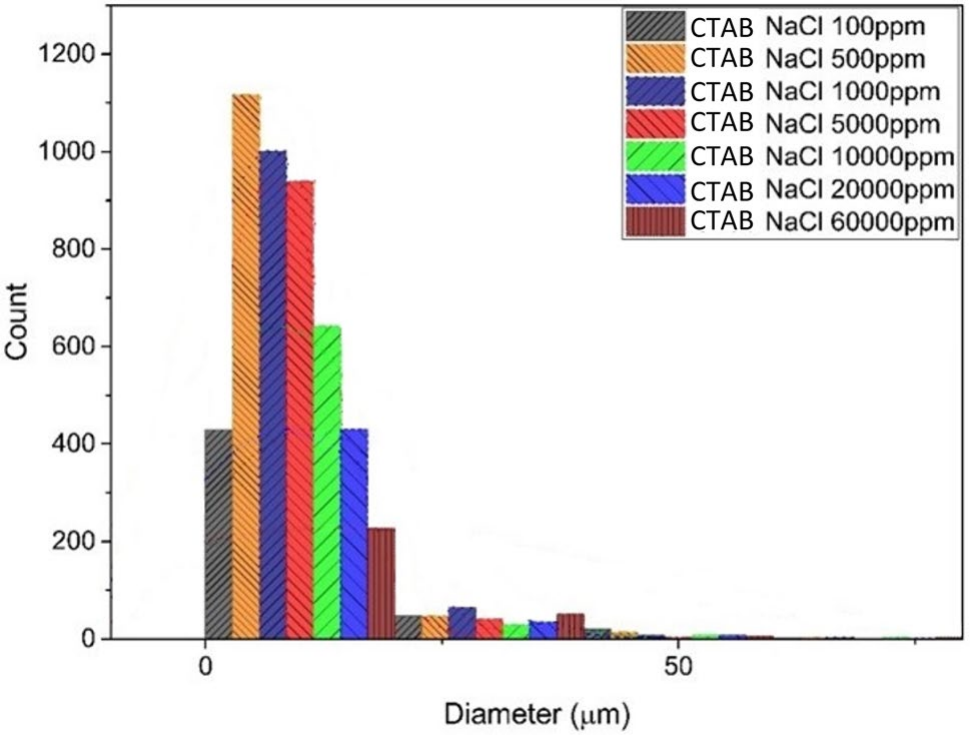
Droplet size distribution diagram of CTAB surfactant emulsion and NaCl salt.

Figure 10 shows the stability of emulsion in the presence of NaCl salt and CTAB surfactant. All experiments were conducted with the same surfactant concentration. According to this figure, maximum number of droplets were observed at the size of less than 10 µm which occurs at the 500 ppm concentration of the salt.

Based on Fig. 10, with the increase of salt from 100 to 500 (ppm), the stability of the emulsion is increased. With the addition of salt to the system, the overall stability has decreased and in its optimal state, stability approaches close to the system without salt. With the increase in salinity, the stability of the emulsion formed for this type of surfactant will significantly decrease and become unstable.

In the separation cycle rate of the centrifuge device for the emulsions formed by CTAB surfactant, the highest separation rate is related to the condition that the salt concentration in the system is equal to 500 (ppm). In this concentration, the rate of separation cycle has been measured equal to 1500 rpm, and increasing the amount of salt in the system, the emulsions have gone towards instability so that the rate of separation cycle has reached its minimum level.

In a general conclusion for NaCl salt in both surfactants, it can be said that the stability of the emulsion is maximum at the concentration of 500 (ppm), which has the maximum amount of small droplets and also the maximum rate of the separation period compared to other concentrations.

#### CaCl_2_–AOS system

**Figure 11:**
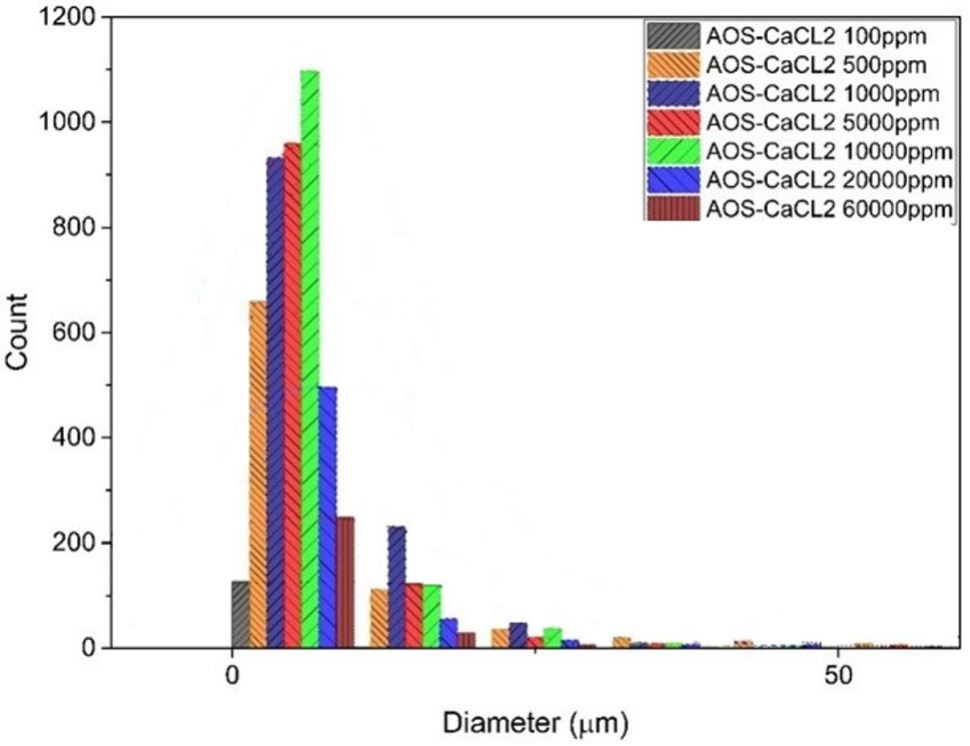
Droplet size distribution diagram of AOS surfactant emulsion and CaCl_2_ salt.

Figure 11 shows the stability of emulsion in the presence of CaCl_2_ salt and AOS surfactant. All experiments were conducted with the same surfactant concentration. According to this figure, maximum number of droplets were observed at the size of less than 10 µm which occurs at the 10,000 ppm concentration of the salt.

According to Fig. 11, by adding salt in the optimal concentration of surfactant, firstly, the stability of the formed emulsions is greatly reduced, and by increasing the amount of salt from 100 to 10,000 (ppm), the number of small droplets of the formed emulsion has increased, and then from this concentration, the amount of drops has decreased significantly.

In the continuation of the experiments related to the centrifuge device, the separation cycle rates of the formed emulsion phase were determined, which, compared to the salt concentration, showed a maximum rate of 3500 revolutions per minute for the separation of the emulsion formed at a concentration of 10,000 (ppm). By comparing the separation cycle rate of the monovalent salt system and the system that contains calcium chloride salt and surfactant, both of which are at their optimal concentration, it is found that the presence of this divalent ion increases the number of dispersed water droplets in the oil phase and causes that to separate this emulsion, a higher rotation rate is needed.

Therefore, the concentration of 10,000 (ppm) stability of the emulsion, both in terms of the number of dispersed small droplets and the rate of the separation period, is at the maximum for the system containing AOS surfactant and CaCl_2_ salt. Further, with the increase of the salt concentration in the system, the rate of separation period follows a decreasing trend, which shows a decrease in the stability of the formed emulsions. Figure 11 also shows the decrease in the number of small droplets in higher concentrations, especially the significant decrease in the concentration of 60,000 (ppm).

#### CaCl_2_–CTAB system

**Figure 12:**
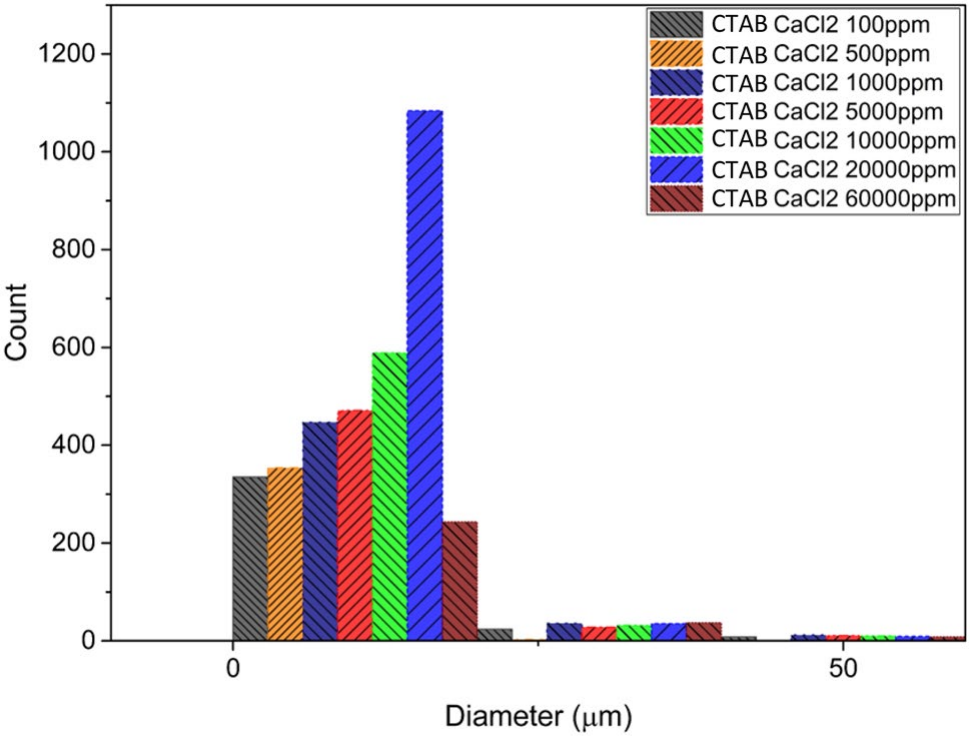
Droplet size distribution diagram of CTAB surfactant emulsion and CaCl_2_ salt.

Figure 12 shows the stability of emulsion in the presence of CaCl_2_ salt and CTAB surfactant. All experiments were conducted with the same surfactant concentration. According to this figure, maximum number of droplets were observed at the size of less than 10 µm which occurs at the 20,000 ppm concentration of the salt.

To investigate the effect of calcium chloride on the formation and stability of the emulsion in the condition that CTAB surfactant is added to the system, it is added to the system in the same way in 7 different concentrations and microscopic photography is done from the samples taken. Figure 12 shows how the system droplets are distributed for this salt.

Based on Fig. 12, the stability of the emulsion from 100 to 10,000 (ppm) followed a uniform increasing method, but at the concentration (ppm) of 60,000, there was a sudden change in this stability, which caused a noticeable increase in the number of small droplets of the dispersed phase. After this concentration, the degree of stability experiences a drastic change and decreases drastically, and the emulsion becomes almost unstable.

Centrifuge tests for this system also show that according to the rotation rate, with increasing stability, there has been an increase in the general state, and in the system that has (ppm) 20,000, this amount has reached the maximum (rotation rate of 2000 revolutions per minute) and after this concentration, the level of stability has reached its minimum level.

#### MgCl_2_–AOS system

**Figure 13:**
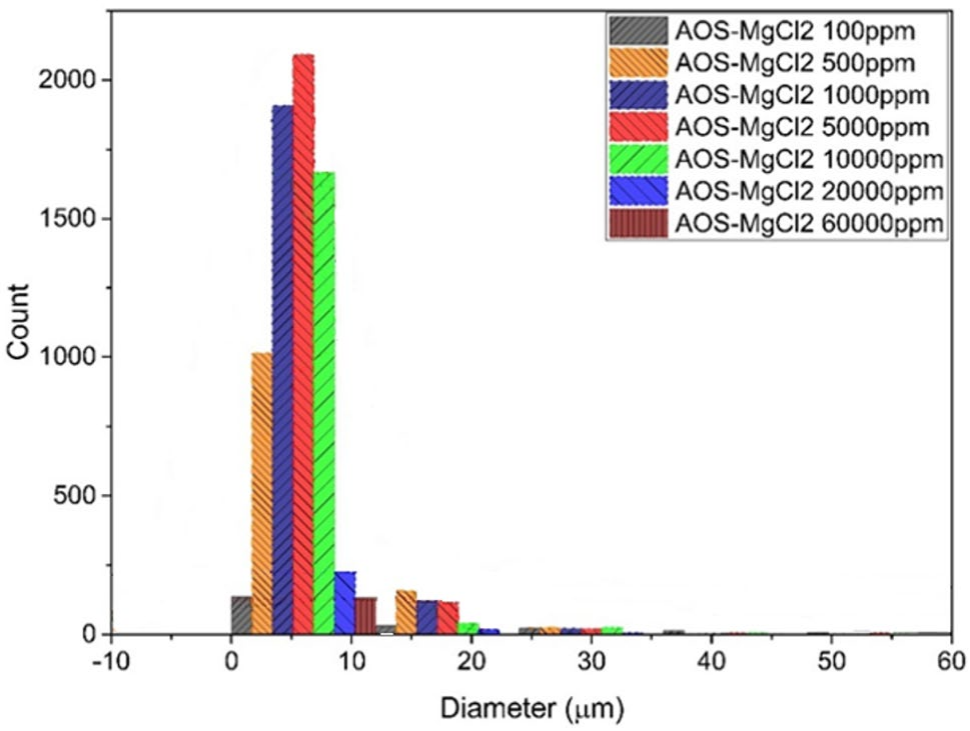
Droplet size distribution diagram of AOS surfactant emulsion and MgCl_2_ salt.

Figure 13 shows the stability of emulsion in the presence of MgCl_2_ salt and AOS surfactant. All experiments were conducted with the same surfactant concentration. According to this figure, maximum number of droplets were observed at the size of less than 10 µm which occurs at the 5000 ppm concentration of the salt.

By adding MgCl_2_ salt to the water phase, initially, the number of small droplets in the formed emulsion is significantly reduced and due to the presence of doubly positive Mg ions, the surfactants in the system be pulled into the water phase, which causes the contact with water droplets and disperse the aqueous phase into the normal heptane phase. By increasing the salt concentration in the system, a process similar to the Salt in effect has occurred, which increases the number of surfactants in the aqueous phase and finally the number of dispersed water droplets in the normal heptane phase. Further, by increasing the concentration of salts in the system, it causes the interaction between the surfactant particles and the salt, and as a result, the ability of the surfactants on the surface between the two phases to separate the drops of the aqueous phase and disperse it in the continuous oil phase is lost.

Adding MgCl_2_ salt to a certain amount causes the asphaltene to be drawn to the surface between water and oil and increases the stability of the emulsion, or in other words, makes the droplets smaller, and after a certain concentration, this effect is reversed. Due to the presence of doubly positive Mg ions, the surfactants in the system are drawn into the aqueous phase, which causes them to come into contact with water droplets and disperse the aqueous phase into the normal heptane phase. By increasing the salt concentration in the system, a process similar to the Salt in effect has occurred, which increases the number of surfactants in the aqueous phase and finally the number of dispersed water droplets in the normal heptane phase.

According to Fig. 13, by increasing the amount of salt in the system from 100 to 5000 (ppm), the stability of the emulsion has increased. After the concentration of 5000, with the increase of ions, the emulsion became unstable and the number of small droplets decreased significantly. The concentration of 5000 (ppm) can be considered the optimal concentration in which the number of small droplets is maximum.

The separation cycle rate for emulsions formed in different concentrations also increased by increasing the amount of salt in the system up to (ppm) 5000, the cycle rate increased up to 4500 rpm and at higher concentrations, this rate increased due to the decrease in the stability of the emulsion has followed a decreasing trend so that it reached a rate of 1500 rpm at a concentration (ppm) of 60,000. Based on centrifuge tests for this system, the concentration stability (ppm) of 5000 is the highest compared to other tested concentrations.

#### MgCl_2_–CTAB system

**Figure 14:**
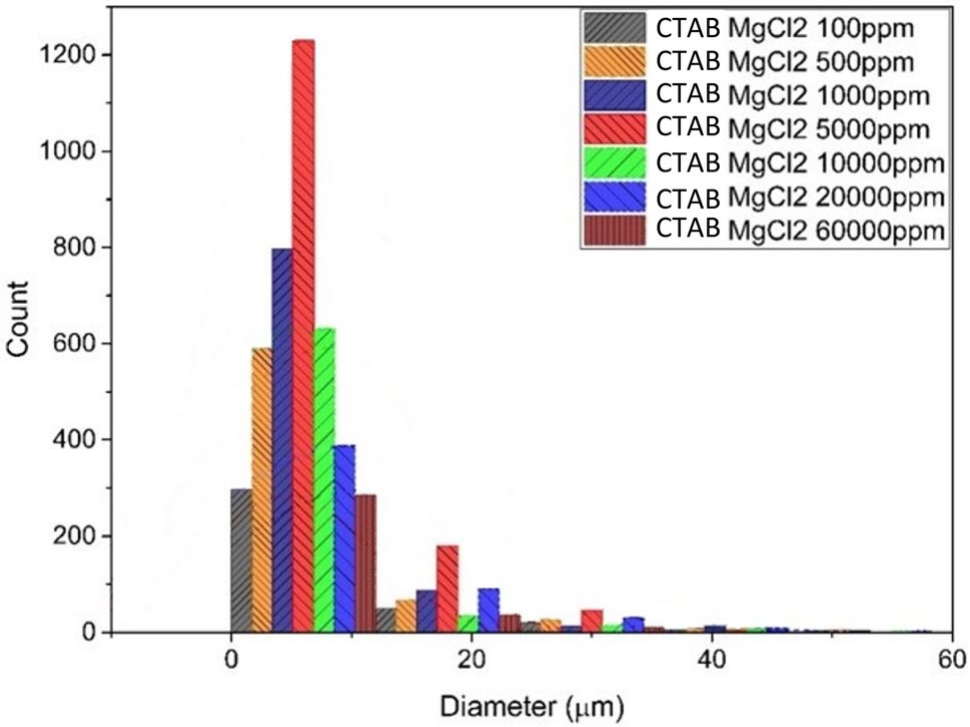
Droplet size distribution diagram of CTAB surfactant emulsion and MgCl_2_ salt.

Figure 14 shows the stability of emulsion in the presence of MgCl_2_ salt and AOS surfactant. All experiments were conducted with the same surfactant concentration. According to this figure, maximum number of droplets were observed at the size of less than 10 µm which occurs at the 5000 ppm concentration of the salt.

According to the data presented in Fig. 14, a noticeable decline in emulsion stability is observed in low salt systems. However, as the salt concentration is raised to 5000ppm, the number of small droplets measuring less than 20 micrometers increases, resulting in a heightened level of emulsion stability. Nevertheless, as the salt concentration continues to rise beyond this threshold, the emulsion stability experiences a decline.

On the other hand, a certain number of these drops indicate an increase in the stability of the system compared to a system without salt. This issue is related to the ability of divalent magnesium ions to pull the polar head of surfactants into the aqueous phase and separate the aqueous phase. In Fig. 14, it can be seen that with the increase in magnesium chloride ion concentration, from 100 to 5000 (ppm), the stability of the formed emulsion increased and the number of drops increased, but with the increase of salt in the system, this stability has the opposite trend. It has progressed and caused a reverse trend in the direction of reducing stability.

In the emulsion centrifuge tests of this system, the increase in rotation rate can be seen with the increase in the stability of the emulsion formed in the system. In the concentration of 100 to 5000 (ppm), it has experienced an increasing trend, and in the concentration (ppm) of 5000, it reaches its maximum, which is equal to 2500 rpm. By increasing the amount of salt in the system, the stability of the emulsion has decreased and as a result, the rotation rate has decreased.

In the emulsion containing AOS surfactant, in the presence of different concentrations of MgCl_2_ salt, the mechanism is such that at a concentration of 5000 ppm the number of small droplets reaches its maximum value of about 2000, using a centrifuge and checking the separation rate for this emulsion In concentrations of 100 ppm, it starts from 1000 rpm until it reaches its maximum value of 4500 rpm at a concentration of 5000 ppm, then with the increase of concentration up to 60,000 ppm, the amount of separation period decreases up to 1500 ppm. That both the image analysis tests of the number and size of the droplet diameter and the centrifuge device indicate the stability of this system at a concentration of 5000 ppm. And the process change in this salt concentration is similar to the mechanisms of salt in effect and salt out effect in the interaction between ion and asphaltene. In this tested set, surfactant plays the same role as asphaltene.

## Conclusion

To compare the strength of AOS and CTAB surfactants, based on the results, it is said that AOS surfactant has more power to form an emulsion, and with the addition of salt and active ions to the system, this stability increases and depending on the type of salt, the quality of the formed emulsion changes. Based on the capacity of active ions, it is said that bivalent ions form a more stable emulsion due to having a higher charge and charge density than the system containing monovalent ions. Based on the results and diagrams of droplet size distribution as well as the flow rate of separation using a centrifuge, the most stable system has AOS surfactant and magnesium chloride salt.

The emulsion system has AOS surfactant along with MgCl_2_ salt in different concentrations, the maximum diameter of fine particles drops is less than 10 µm at a concentration of 5000 (ppm), which is equal to 2200 and with a separation flow rate of 4500 rpm. Also, in the emulsion system with CTAB surfactant along with MgCl_2_ salt in different concentrations, the maximum diameter of fine particles droplets is less than 10 µm at a concentration of 5000 (ppm), which is equal to 1200 and a separation flow rate of 2500 rpm. In terms of stability, the salt containing system is MgCl_2_ > CaCl_2_ > NaCl.

## Data Availability

All data generated or analysed during this study are included in this published article.
